# END-OF-LIFE EVENTS AND CAUSES OF DEATH IN DANISH LONG-LIVED FEMALE SIBLINGS

**DOI:** 10.1093/geroni/igac059.1555

**Published:** 2022-12-20

**Authors:** Angeline Galvin, Svetlana Ukraintseva, Konstantin Arbeev, Mary Feitosa, Anne Newman, Kaare Christensen

**Affiliations:** University of Southern Denmark, Odense, Syddanmark, Denmark; Duke University, Durham, North Carolina, United States; Duke University, Durham, North Carolina, United States; Washington University, St Louis, Missouri, United States; University of Pittsburgh, Pittsburgh, Pennsylvania, United States; Southern Denmark University, Odense, Syddanmark, Denmark

## Abstract

Long-lived siblings have better health and survival compared to “sporadic” long-lived individuals, but it is unknown whether they also differ in end-of-life events and causes of death. Deceased Danish long-lived female siblings (n=833, mean age at death=95.6) were identified through national health registers compared to controls matched on sex, year-of-birth, and year-of-death. End-of-life events (hospitalizations, emergency room visits, medication within the five years before death) and causes of death were analyzed using linear models with fixed effects and multinomial logistic models, respectively. End-of-life events and causes of death were not statistically significantly different between long-lived female siblings and “sporadic” long-lived individuals. However, long-lived female siblings presented non-significant higher risk of ischemic heart disease and cancer – and lower risk of mental diseases and accidents. The analyses will be extended to include men, a longer follow-up, and focus on dementia in the last years of life.

